# Supplementary figures and images for: Phylogenomic timetree-calibrated speciation clocks for Caenorhabditis nematodes reveal slow but disproportionate accumulation of post-zygotic reproductive isolation
Source: PLoS Genet. 2025 Sep 11;21(9):e1011852. doi: 10.1371/journal.pgen.1011852 (PMC12440214; doi:10.1371/journal.pgen.1011852)

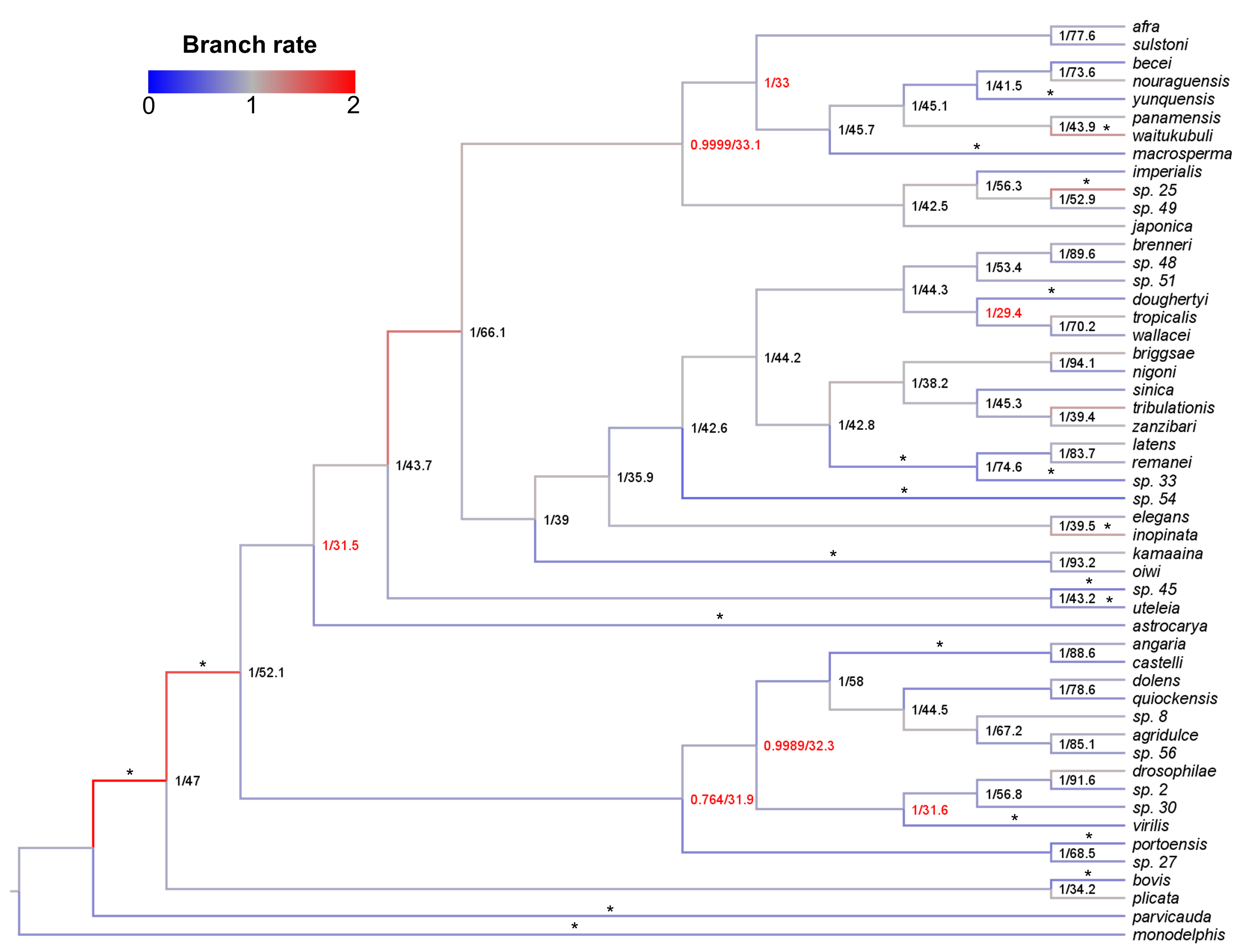

Supplement: S1 Fig — Numerical labels on each node give the posterior probability (out of 1) followed by the site concordance factor (out of 100). Nodes with low statistical support (posterior probability < 1) or high discordance (site concordance factor < 33.3%) are labeled in red. The color of each branch gives its relative substitution rate under the relaxed clock model, with higher rates indicated by shades of red and slower rates indicated by shades of blue. Branches with a “*” have a 95% Highest Posterior Density interval for the estimated branch rate that does not overlap with 1. (TIF) [file pgen.1011852.s007.tif]

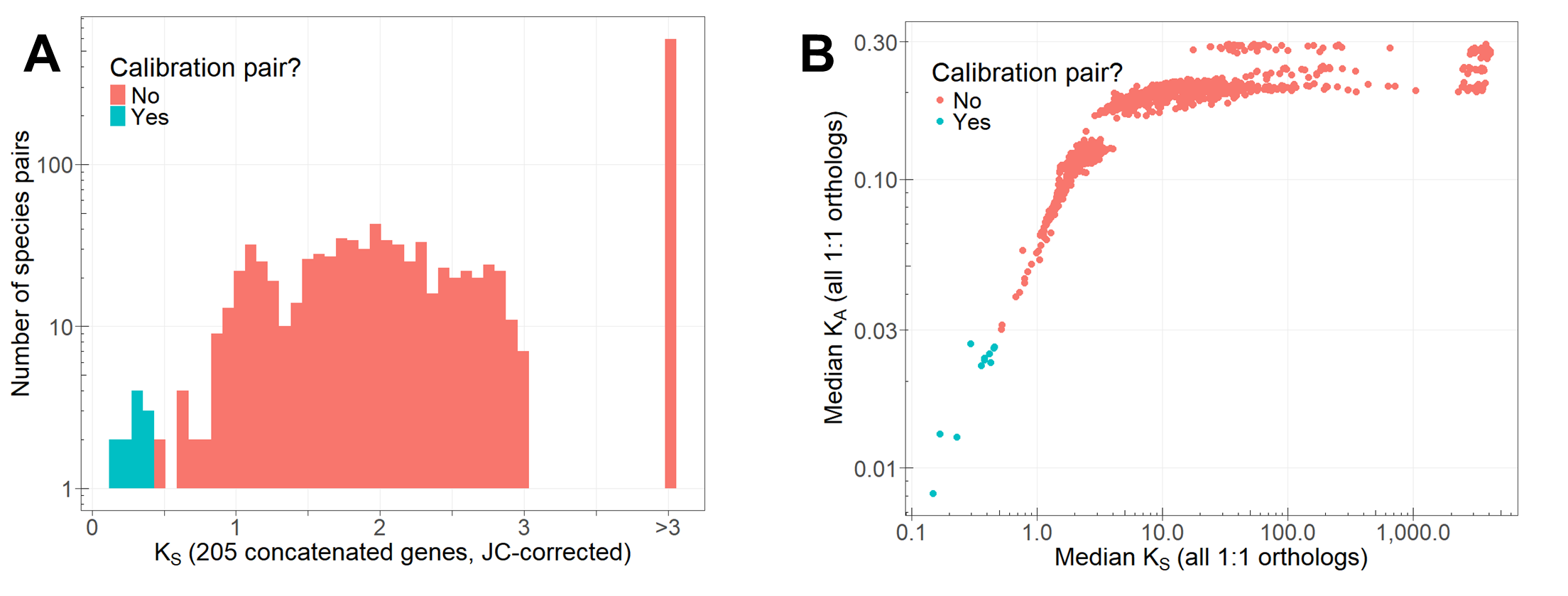

Supplement: S2 Fig — The 11 species pairs chosen for use in calibration include: C. sp. 56 – C. agridulce, C. remanei – C. latens, C. nigoni – C. briggsae, C. drosophilae – C. sp. 2, C. brenneri – C. sp. 48, C. angaria – C. castelli, C. oiwi – C. kamaaina, C. dolens – C. quiockensis, C. becei – C. nouraguensis, C. sp. 56 – C. sp. 8, and C. agridulce – C. sp. 8. B. Comparison of median KS and median KA between all 1275 pairs of the 51 species used for divergence time estimation, calculated with FitMG94 based on all 1:1 orthologs between each species pair. (TIF) [file pgen.1011852.s008.tif]

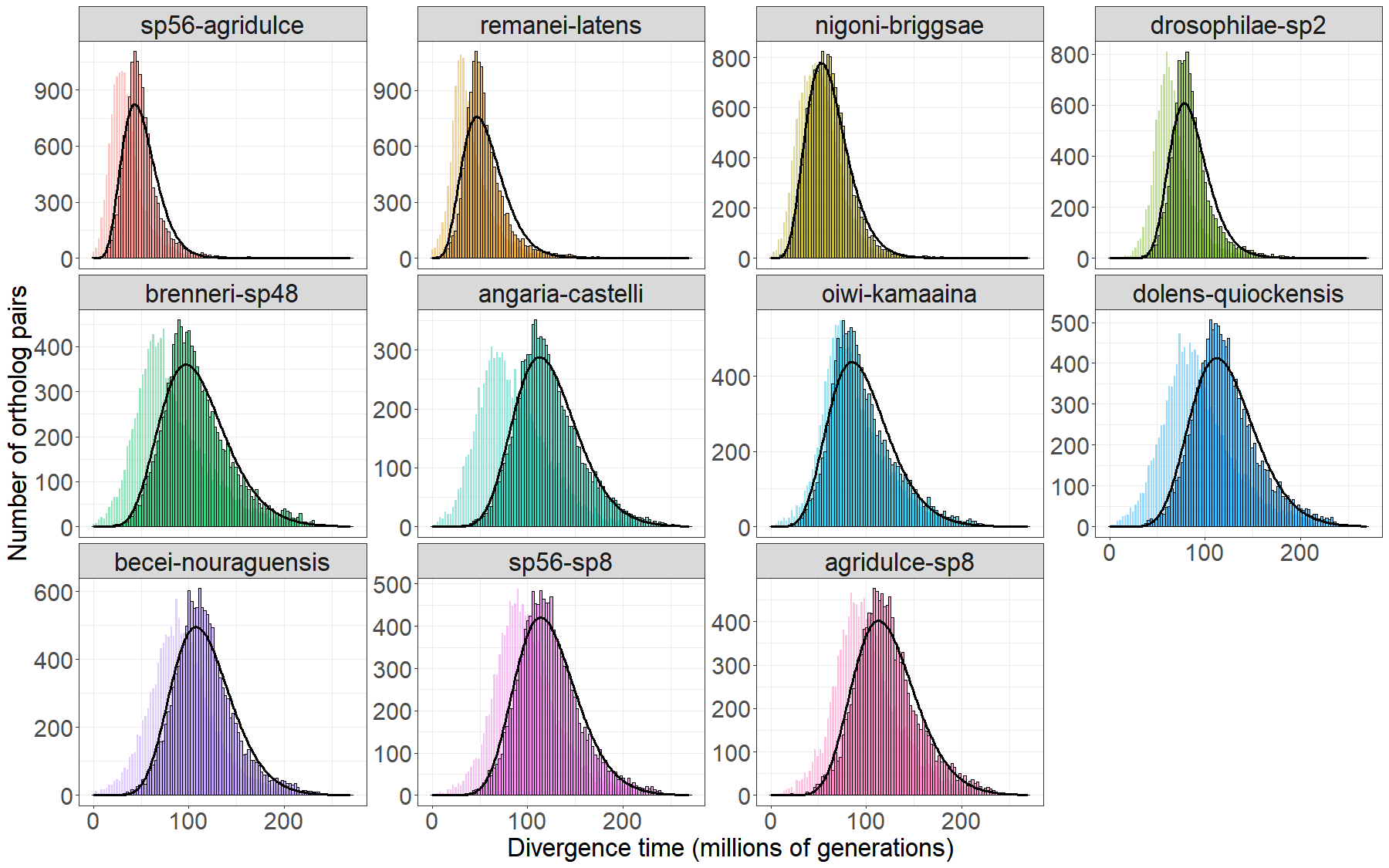

Supplement: S3 Fig — Black curves on each panel show the density of the prior distribution that was given to BEAST for each species pair (for the common ancestor of C. sp. 8, C. sp. 56, and C. agridulce, only the C. sp. 8 – C. sp. 56 pair was used), based on fits to a gamma distribution (except the C. drosophilae – C. sp. 2 pair, for which a log-normal distribution better fit the data based on log-likelihood). (TIF) [file pgen.1011852.s009.tif]

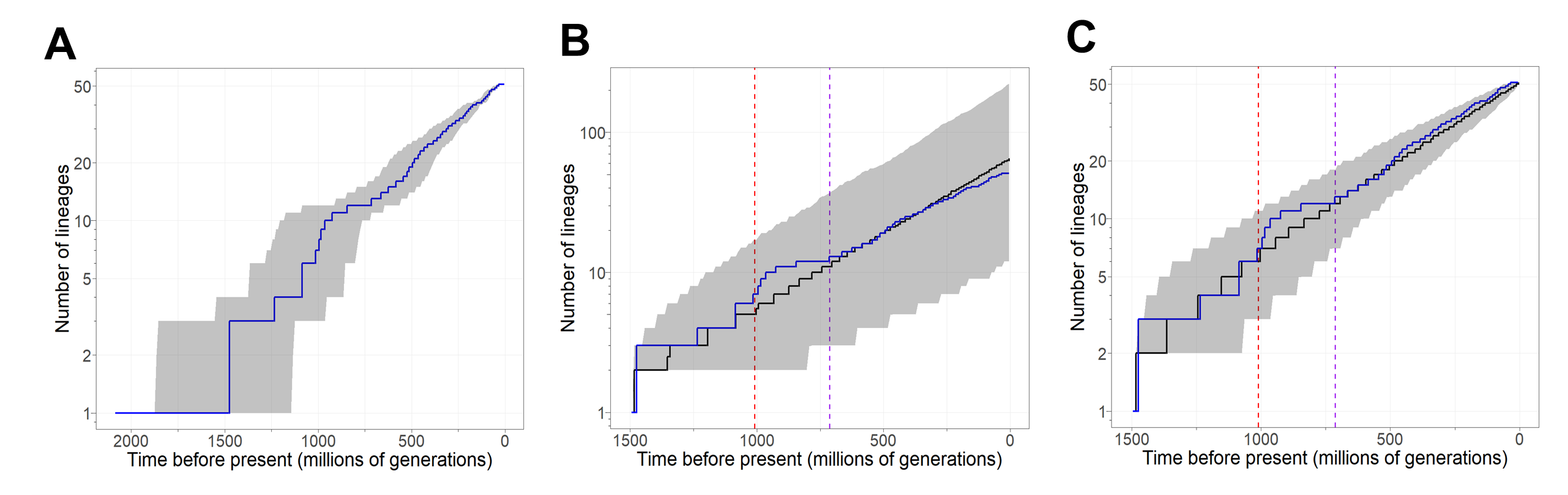

Supplement: S4 Fig — Blue lines in all panels indicate the cumulative observed number of lineages through time for the 51-species phylogeny. B. Lineage-through-time plot for 1000 random phylogenies with the same total age as our primary phylogeny, but allowing any final number of tips given the estimated birth rate. Phylogenies were generated under the birth rate (λ = 0.0248 per lineage per 10 million generations) estimated from our primary phylogeny when assuming a species sampling fraction of 59% (51/86). Shaded intervals give the 95% upper and lower quantiles around the median, based on the 1000 random trees. C. Lineage-through-time plot for 1000 random phylogenies, constrained to have the same total age and final number of tips (51) as our primary phylogeny. Phylogenies were generated under the birth rate (λ = 0.0191 per lineage per 10 million generations) estimated from our primary phylogeny when assuming a species sampling fraction of 100%. Shaded intervals give the 95% upper and lower quantiles around the median, based on the 1000 random trees. Vertical dashed lines in B-C indicate the ages of the Elegans supergroup (purple) and the basal clade defined by the most recent common ancestor of C. angaria and C. portoensis (red). (TIF) [file pgen.1011852.s010.tif]

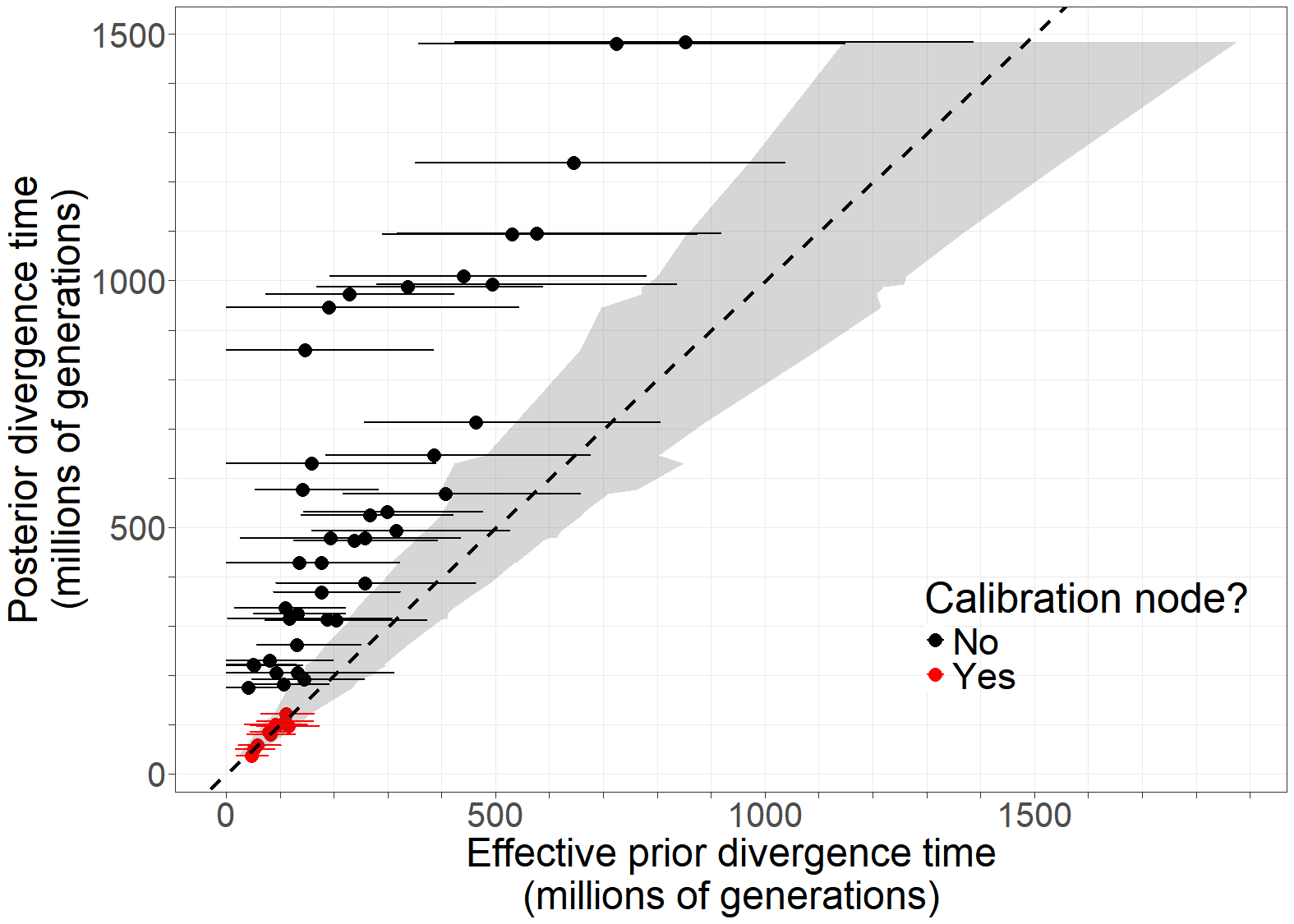

Supplement: S5 Fig — Shaded interval gives the 95% Highest Posterior Density (HPD) interval around the posterior dates. Error bars around each point give the 95% HPD interval of the effective prior dates. Dashed line gives the 1:1 line. Of the 40 non-calibration nodes, 38 nodes have a mean effective prior date that falls outside of the 95% HPD interval of the corresponding posterior, with 13 nodes having a 95% HPD interval around the effective prior that does not overlap with the 95% HPD interval of the corresponding posterior. (TIF) [file pgen.1011852.s011.tif]

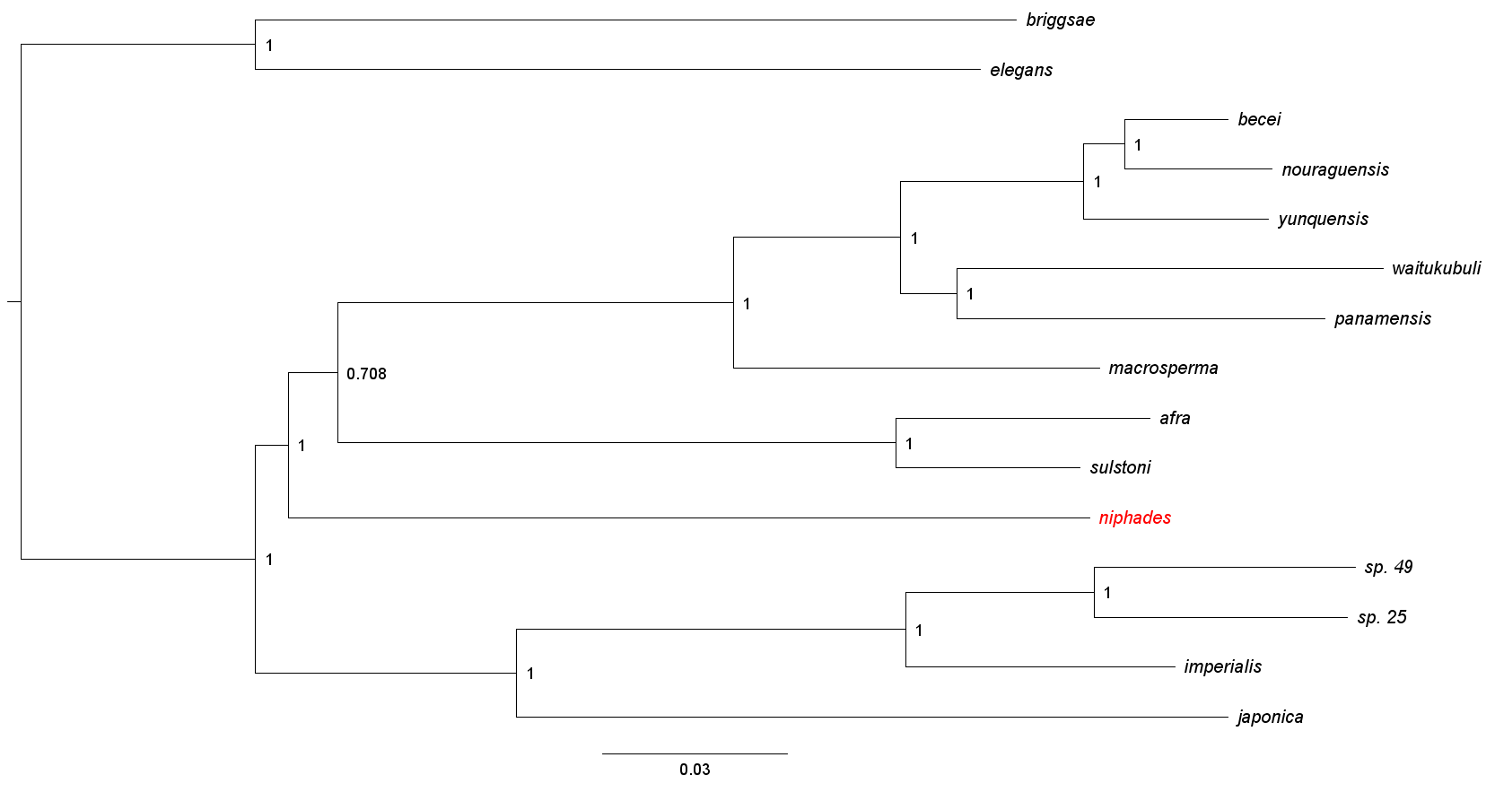

Supplement: S6 Fig — Node labels indicate posterior probabilities. Branch lengths are in units of amino acid substitutions per site. (TIF) [file pgen.1011852.s012.tif]

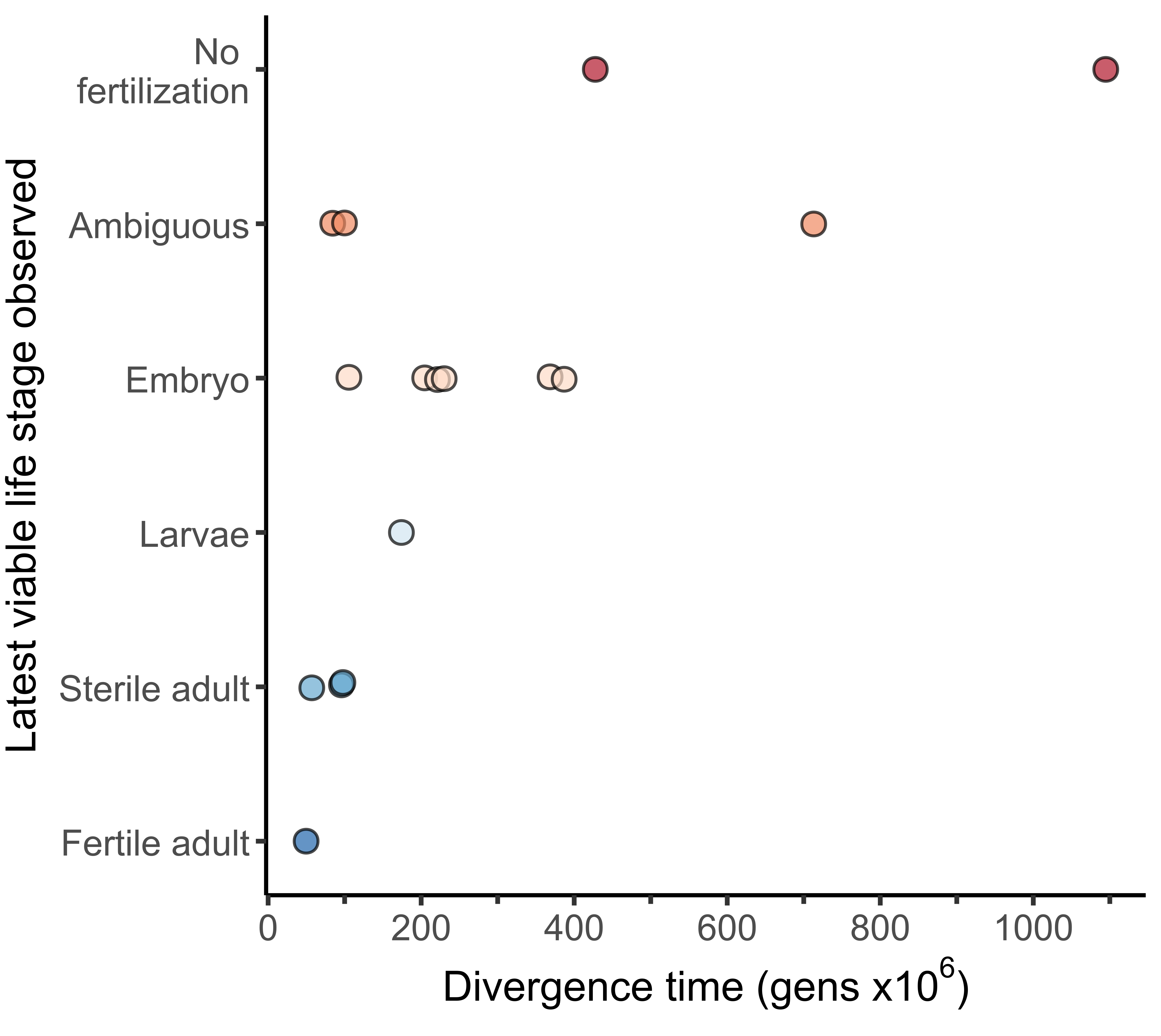

Supplement: S8 Fig — In cases where reciprocal cross directions produced F1 offspring that reached different terminal life stages, the latest stage is indicated. (TIF) [file pgen.1011852.s014.tif]

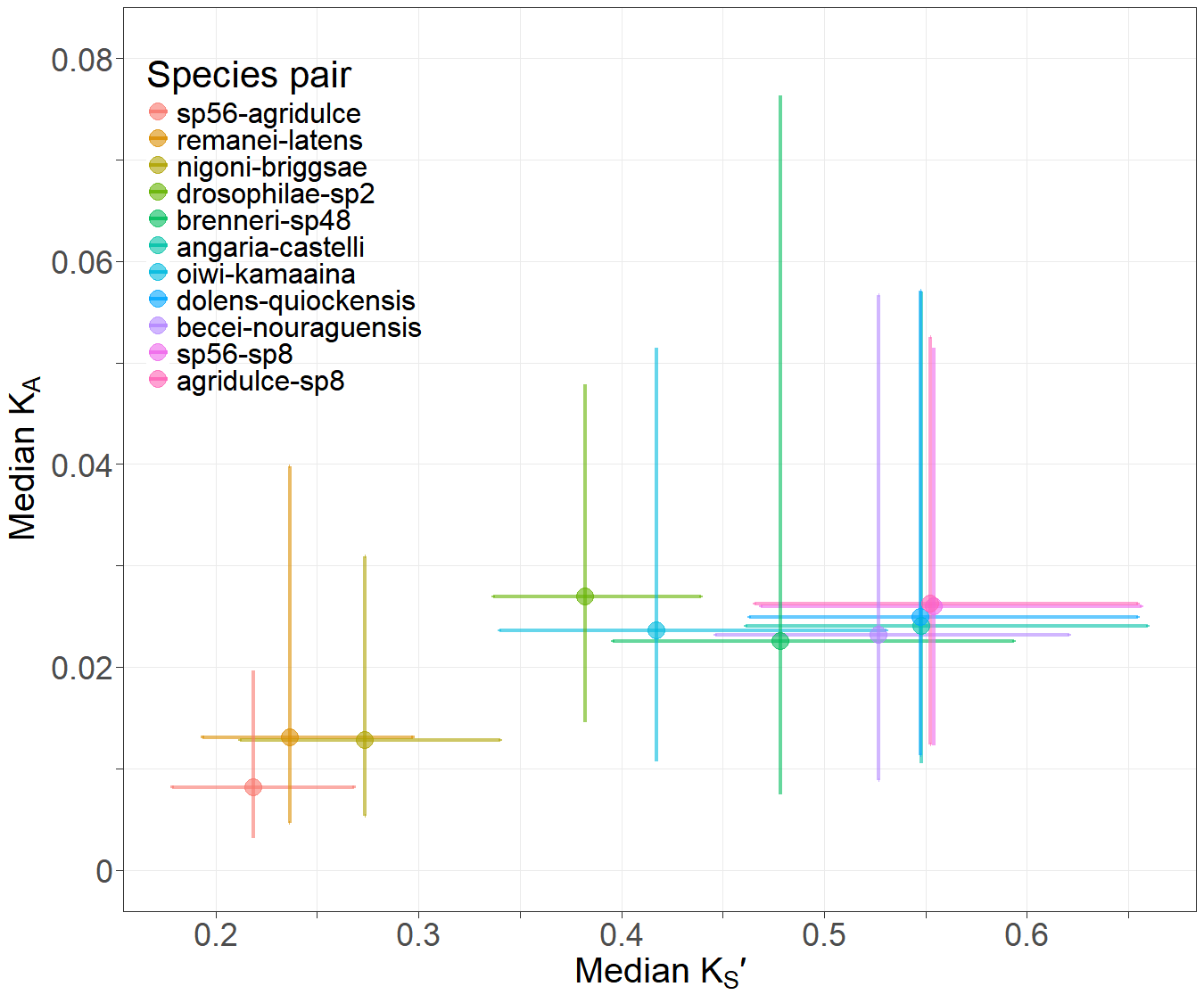

Supplement: S9 Fig — Number of 1:1 ortholog pairs per species pair ranges from 9144 to 15,341 for KA (median 13,020) and 8774–15,204 for KS′ (median 12,605). Error bars indicate interquartile ranges. (TIF) [file pgen.1011852.s015.tif]
