# Supplementary material for: Phylogenomic timetree-calibrated speciation clocks for Caenorhabditis nematodes reveal slow but disproportionate accumulation of post-zygotic reproductive isolation
Source: PLoS Genet. 2025 Sep 11;21(9):e1011852. doi: 10.1371/journal.pgen.1011852 (PMC12440214; doi:10.1371/journal.pgen.1011852)
Supplement: S1 Text — Supporting information including list of supplementary tables and supplementary figures. (PDF) [file pgen.1011852.s016.pdf]

S1 Text

Supporting information for Fusca et al. "Phylogenomic timetree-calibrated speciation clocks for *Caenorhabditis* nematodes reveal slow but disproportionate accumulation of post-zygotic reproductive isolation"

Supplementary Tables in files "S1_Table.xlsx", "S2_Table.xlsx", "S3_Table.xlsx", "S4_Table.xlsx", "S5_Table.xlsx", "S6_Table.xlsx".

Supplementary Table S1: Sources of the genome/transcriptome assemblies and gene annotation files for the 51 *Caenorhabditis* species used in our primary divergence time estimates, as well as *C. auriculariae* and *C. niphades*. All data from the Caenorhabditis Genomes Project are also available at https://doi.org/10.5281/zenodo.12633738.

Supplementary Table S2: For every pair of the 51 *Caenorhabditis* species used in our primary divergence time estimates, median K_A and median K_S across 1:1 orthologs, the number of 1:1 ortholog pairs used to calculate these medians, and our primary divergence time estimates based on 205 single-copy orthologs (i.e. times shown in Fig. 1, in units of millions of generations).

Supplementary Table S3: The values of K_S (both uncorrected and corrected, i.e. K_S') and ENC for every pair of 1:1 orthologs used to estimate calibration time priors for our 11 calibration species pairs (i.e. 1:1 ortholog pairs with uncorrected $K_S \leq 1$).

Supplementary Table S4: For every species pair involving either *C. auriculariae* or *C. niphades* in comparison to one of our 51 primary species (or to each other), median K_A and median K_S across 1:1 orthologs, the number of 1:1 ortholog pairs used to calculate these medians, and the estimate of divergence time based on the median K_A value and our K_A–divergence time regression.

Supplementary Table S5: Phylogenetically-independent species pairs used to generate logistic fits for pre-zygotic, post-zygotic and F1 reproductive isolation and for regression analysis on latest viable stage of F1 hybrids.

Supplementary Table S6: Logistic function fits to components of reproductive isolation (RI) when conspecific divergence times are set to 0 or excluded.

Supplementary Figures

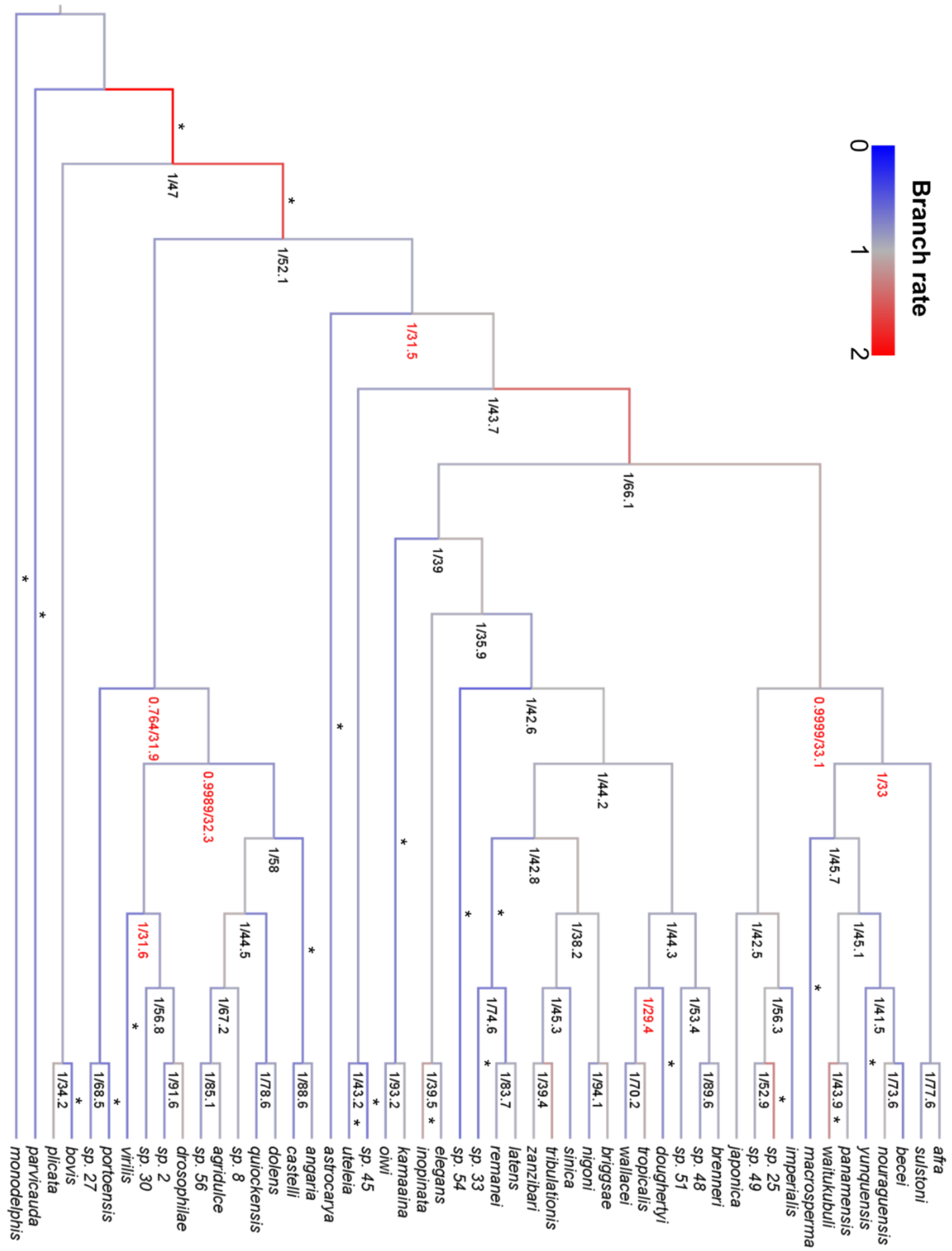

Figure S1. Topology of the ASTRAL species tree used for our primary divergence time estimates (Figure 1). Numerical labels on each node give the posterior probability (out of 1) followed by the site concordance factor (out of 100). Nodes with low statistical support (posterior probability < 1) or high discordance (site concordance factor < 33.3%) are labeled in red. The color of each branch gives its relative substitution rate under the relaxed clock model,

with higher rates indicated by shades of red and slower rates indicated by shades of blue. Branches with a “*” have a 95% Highest Posterior Density interval for the estimated branch rate that does not overlap with 1.

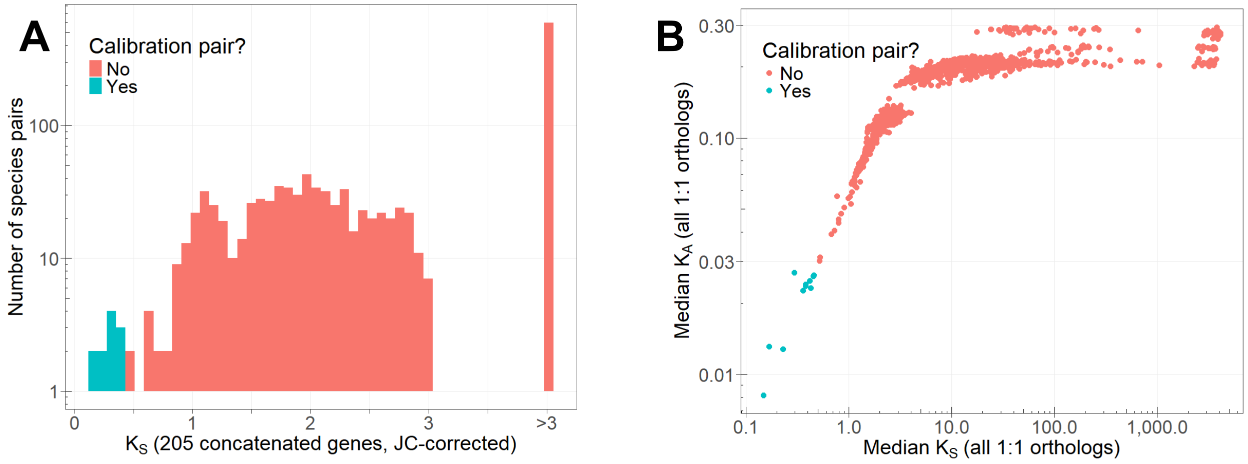

Figure S2. A. Histogram of Jukes-Cantor corrected values of synonymous-site substitution rate (K_S) between all 1275 pairs of the 51 species used for divergence time estimation, calculated in MEGA based on the concatenation of the 205 single-copy orthologs. The 11 species pairs chosen for use in calibration include: *C.* sp. 56 – *C. agridulce*, *C. remanei* – *C. latens*, *C. nigoni* – *C. briggsae*, *C. drosophilae* – *C.* sp. 2, *C. brenneri* – *C.* sp. 48, *C. angaria* – *C. castelli*, *C. oiwi* – *C. kamaaina*, *C. dolens* – *C. quiockensis*, *C. becei* – *C. nouraguensis*, *C.* sp. 56 – *C.* sp. 8, and *C. agridulce* – *C.* sp. 8. **B.** Comparison of median K_S and median K_A between all 1275 pairs of the 51 species used for divergence time estimation, calculated with FitMG94 based on all 1:1 orthologs between each species pair.

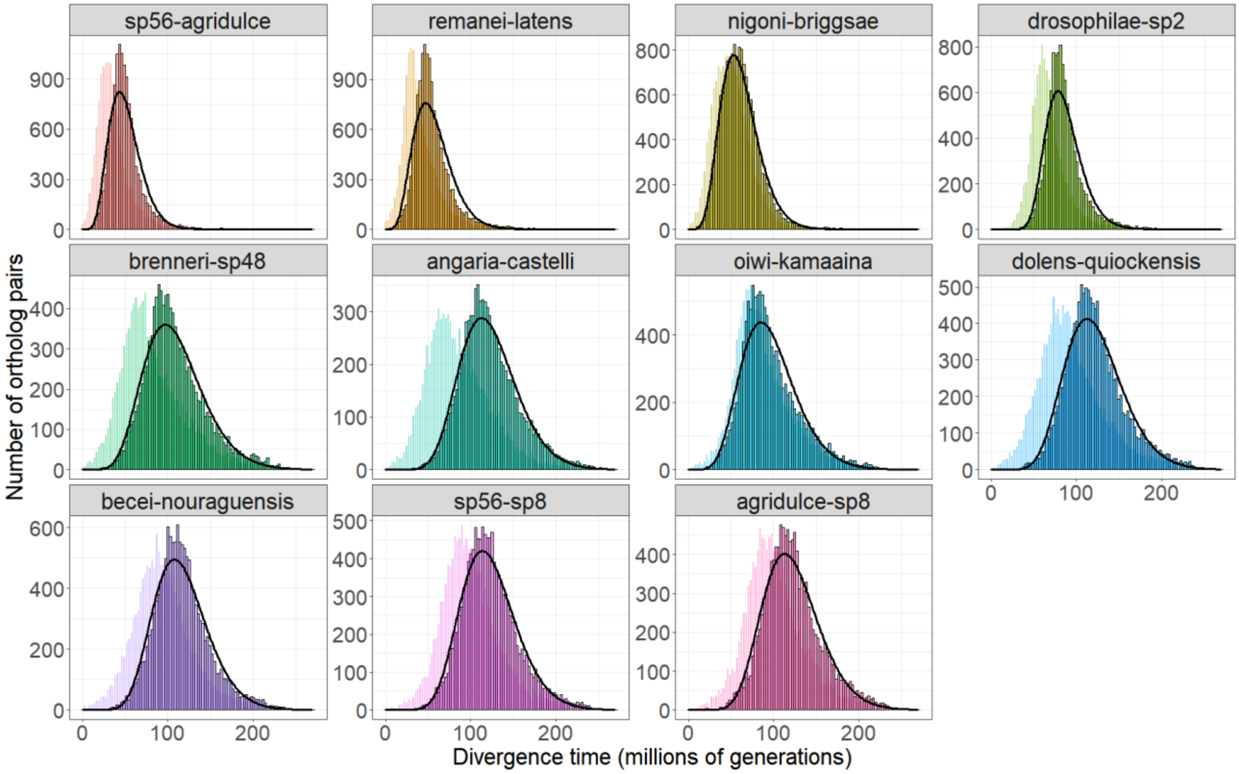

Figure S3. Distributions of divergence times for each calibration species pair, calculated by converting ENC-corrected values of K_S' (foreground, black-outlined bars) or uncorrected values of K_S (background, lighter bars) for 1:1 orthologs to divergence times using the strict molecular clock with an experimentally-derived estimate of mutation rate. Black curves on each panel show the density of the prior distribution that was given to BEAST for each species pair (for the common ancestor of *C.* sp. 8, *C.* sp. 56, and *C. agridulce*, only the *C.* sp. 8 – *C.* sp. 56 pair was used), based on fits to a gamma distribution (except the *C. drosophilae* – *C.* sp. 2 pair, for which a log-normal distribution better fit the data based on log-likelihood).

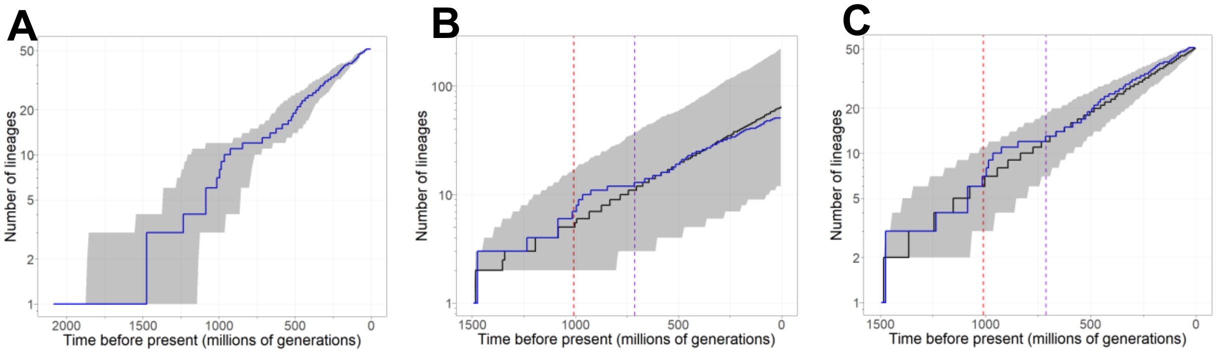

Figure S4. A. Lineage-through-time plot for the *Caenorhabditis* genus. Shaded intervals give the 95% upper and lower quantiles around the median, based on the posterior distributions of divergence times calculated by BEAST. Blue lines in all panels indicate the cumulative observed number of lineages through time for the 51-species phylogeny. **B.** Lineage-through-time plot for 1000 random phylogenies with the same total age as our primary phylogeny, but allowing any final number of tips given the estimated birth rate. Phylogenies were generated under the birth rate (λ=0.0248 per lineage per 10 million generations) estimated from our primary phylogeny when assuming a species sampling fraction of 59% (51/86). Shaded intervals give the 95% upper and lower quantiles around the median, based on the 1000 random trees. **C.** Lineage-through-time plot for 1000 random phylogenies, constrained to have the same total age and final number of tips (51) as our primary phylogeny. Phylogenies were generated under the birth rate (λ=0.0191 per lineage per 10 million generations) estimated from our primary phylogeny when assuming a species sampling fraction of 100%. Shaded intervals give the 95% upper and lower quantiles around the median, based on the 1000 random trees. Vertical dashed lines in B-C indicate the ages of the Elegans supergroup (purple) and the basal clade defined by the most recent common ancestor of *C. angaria* and *C. portoensis* (red).

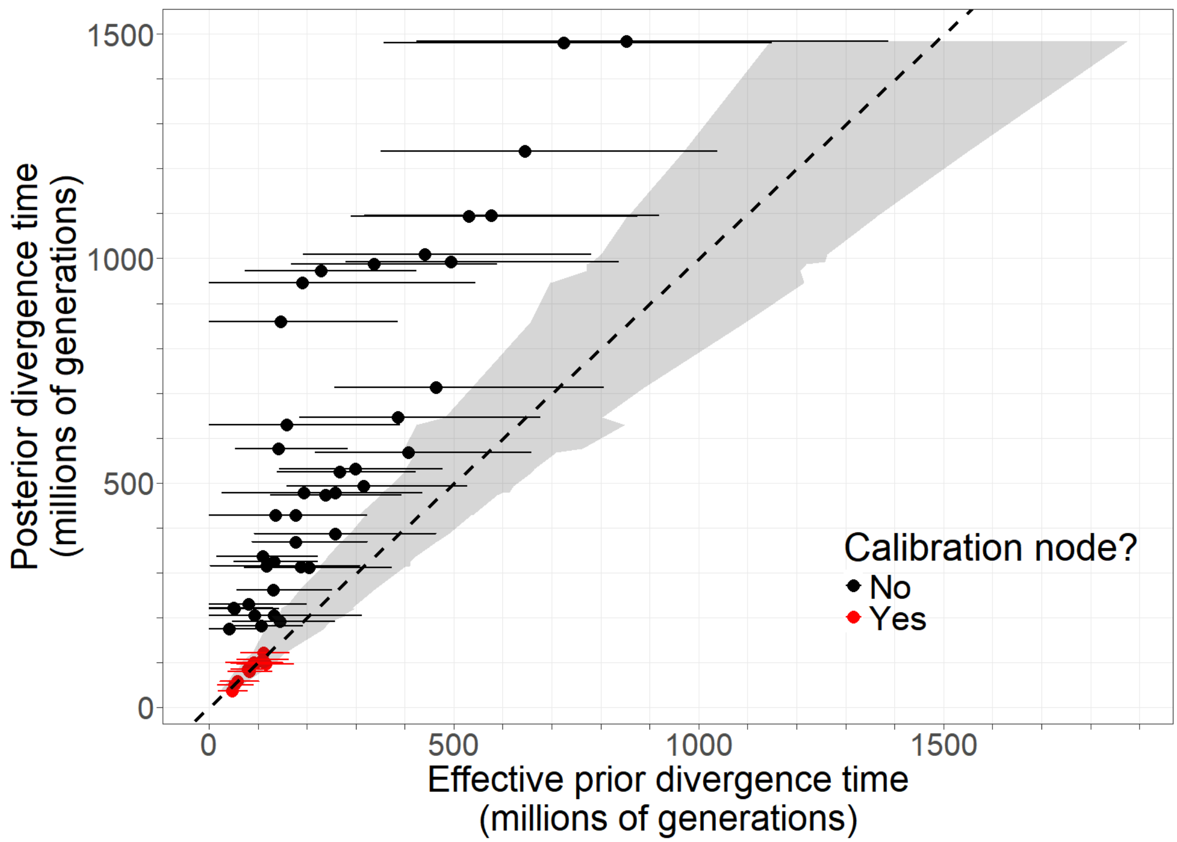

Figure S5. Comparison of divergence time estimates for each species pair between the primary dates based on 205 genes (posteriors) and the dates estimated when ignoring the alignment data (effective priors). Shaded interval gives the 95% Highest Posterior Density (HPD) interval around the posterior dates. Error bars around each point give the 95% HPD interval of the effective prior dates. Dashed line gives the 1:1 line. Of the 40 non-calibration nodes, 38 nodes have a mean effective prior date that falls outside of the 95% HPD interval of the corresponding posterior, with 13 nodes having a 95% HPD interval around the effective prior that does not overlap with the 95% HPD interval of the corresponding posterior.

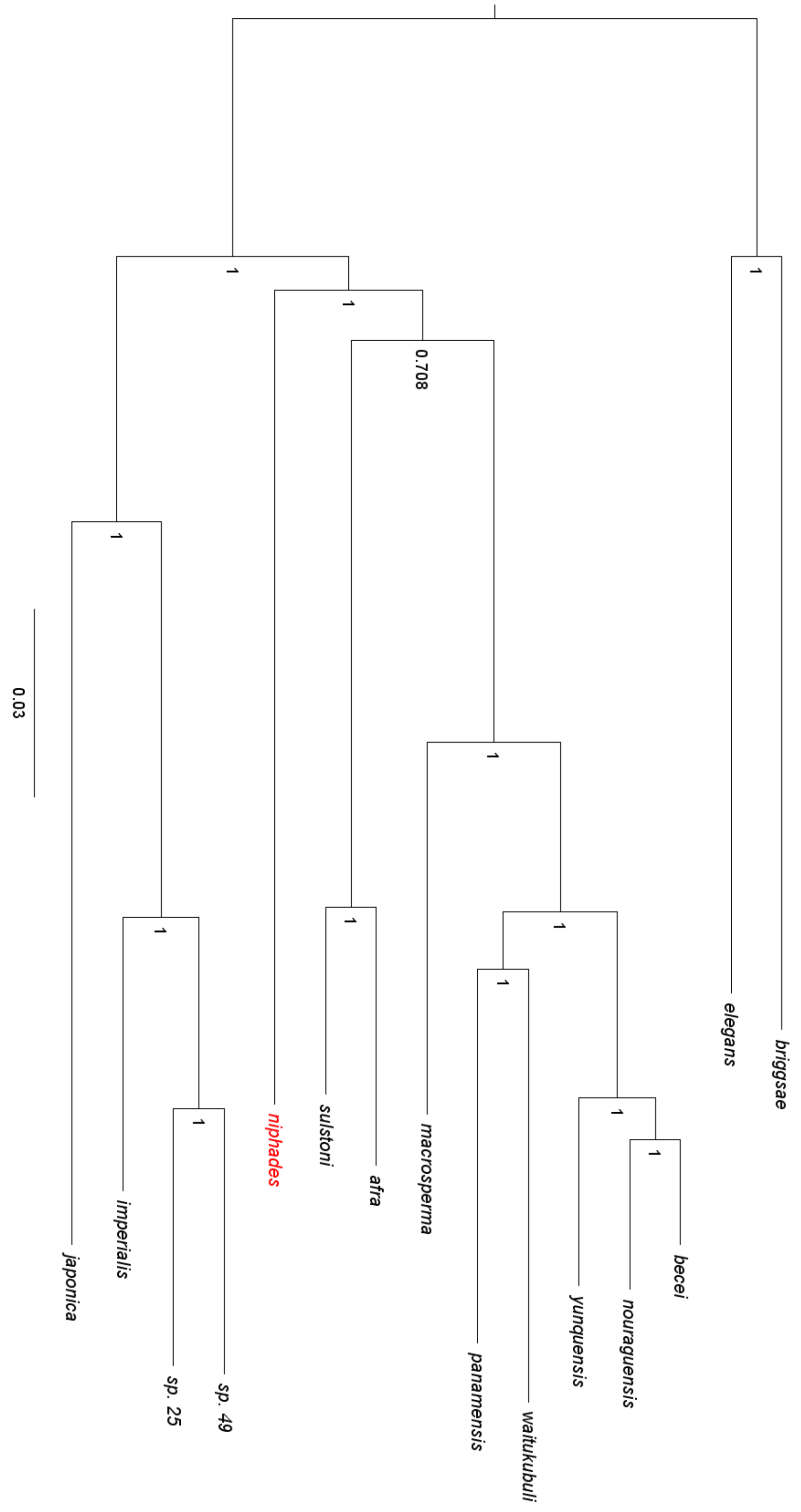

Figure S6. ASTRAL species tree of the Japonica group of species (including *C. niphades*, in red), as well as the outgroups *C. elegans* and *C. briggsae*. Node labels indicate posterior probabilities. Branch lengths are in units of amino acid substitutions per site.

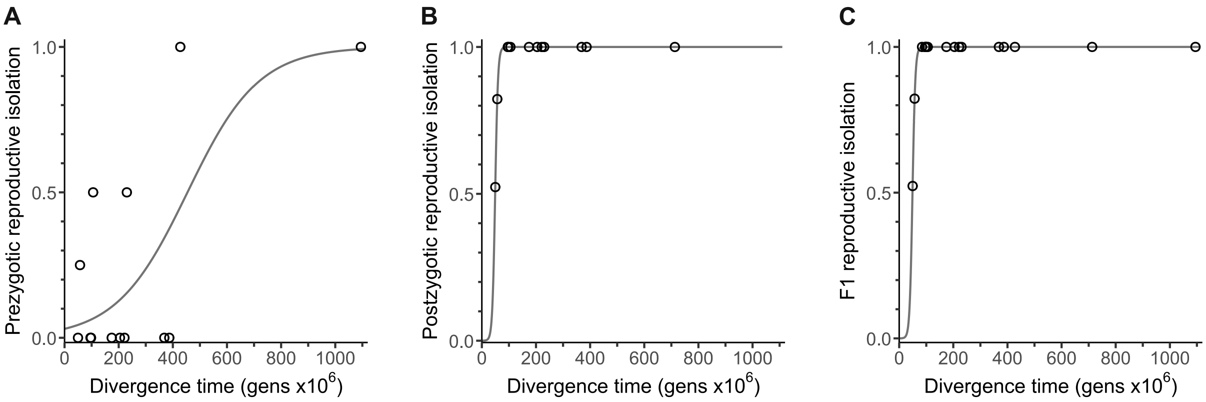

Figure S7. Reproductive isolation clocks generated using 16 phylogenetically independent species pairs (some RI metrics have fewer pairs), with reciprocal cross values averaged for a given pair.

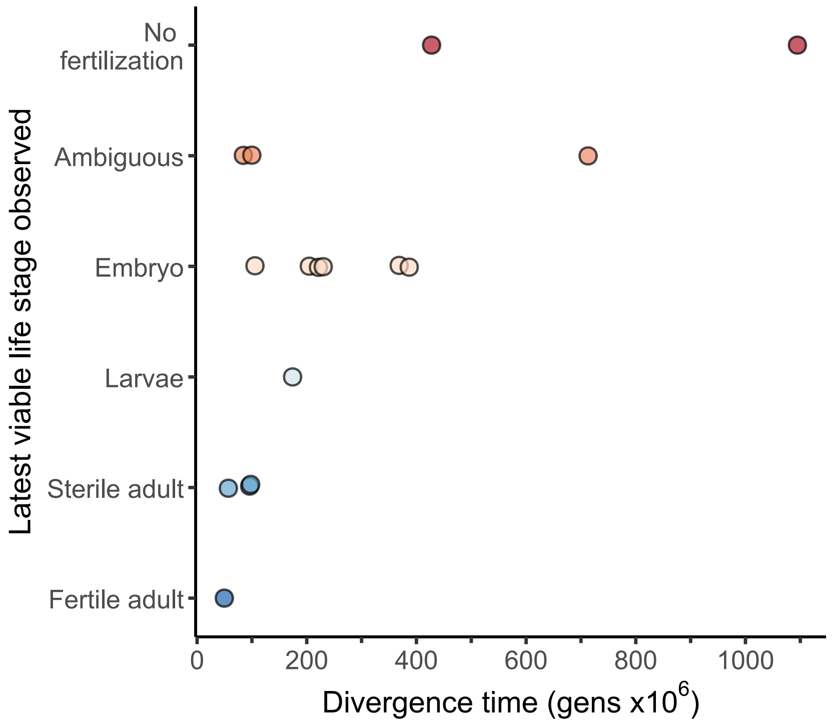

Figure S8. Latest stage reached by F1 offspring of 16 phylogenetically-independent species pairs. In cases where reciprocal cross directions produced F1 offspring that reached different terminal life stages, the latest stage is indicated.

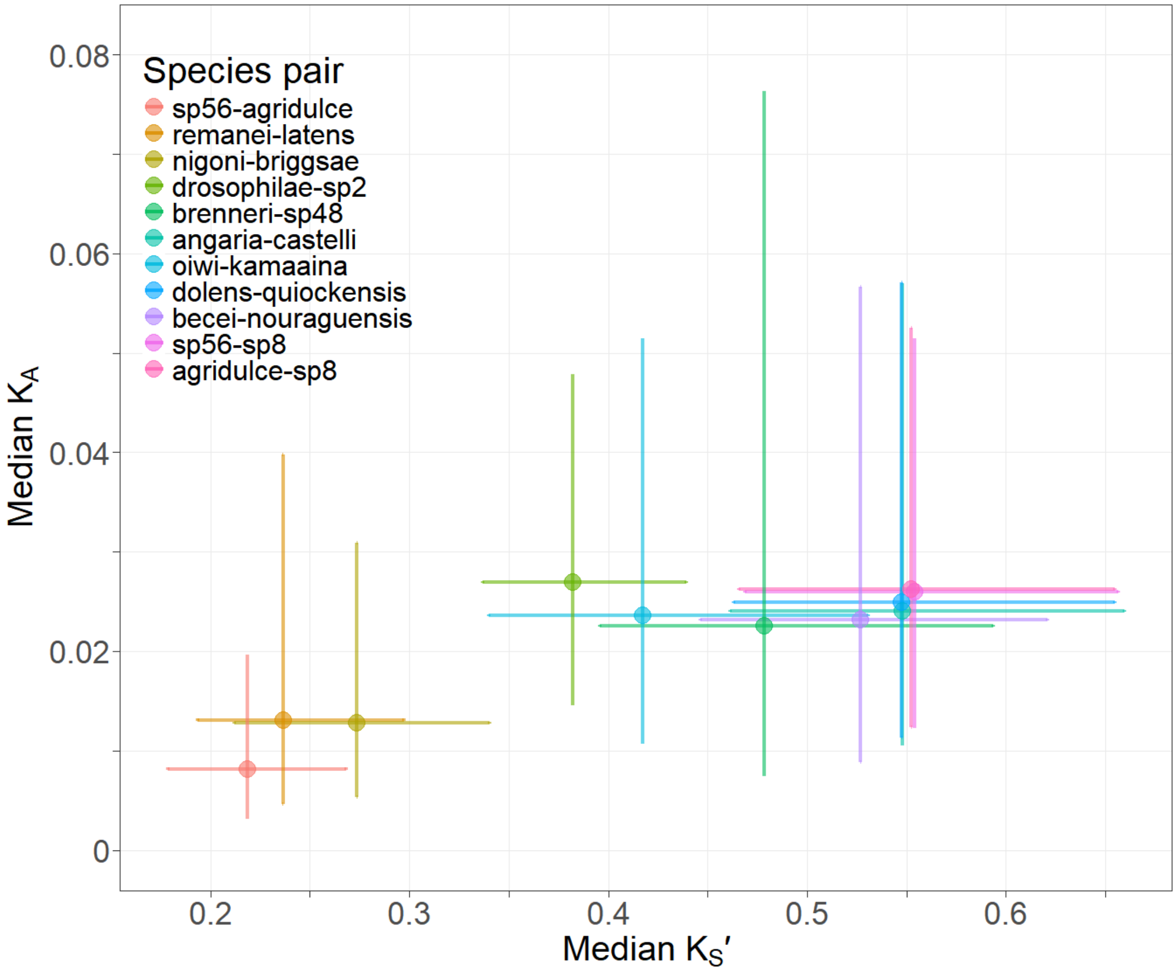

Figure S9. Median divergence across 1:1 orthologs for replacement site differences (K_A) and synonymous site differences corrected for codon usage bias (K_S') for the 11 pairs of *Caenorhabditis* species used for divergence time calibration. Number of 1:1 ortholog pairs per species pair ranges from 9144 to 15,341 for K_A (median 13,020) and 8774 to 15,204 for K_S' (median 12,605). Error bars indicate interquartile ranges.
